# Exploring the Benefits of Assistive Devices Use in Older Adults With Impaired Lower Limb Function

**DOI:** 10.1093/geroni/igaa057.955

**Published:** 2020-12-16

**Authors:** Tai-Te Su, Shannon Mejia

**Affiliations:** 1 University of Illinois at Urbana-Champaign, Urbana, Illinois, United States; 2 University of Illinois at Urbana-Champaign, Champaign, Illinois, United States

## Abstract

Assistive technologies are an essential component of meeting the needs of an aging population. With advancing age, chronic conditions and physiological changes can result in impaired lower limb function among older adults, which may in turn limit their ability to perform daily activities or even walking. Informed by the continuity theory, we conceptualize assistive devices (ADs) as a resource that older adults with mobility limitations can leverage to remain active and mobile in late life. However, evidence remains scarce to determine the extent to which using ADs could create a measurable change in older people’s experienced well-being. Using data from the 2012 wave of the Health and Retirement Study, our study aimed to examine the potential psychological benefits that accompany ADs use in a sample of older adults with at least some limited lower limb mobility (n = 505, 59% female, 85.6% white, age = 75.8 ± 6.7). Results from multiple linear regression showed that although AD use was not directly associated with global well-being, those who used ADs reported more positive experience while walking (b = 0.65, SE = 0.23, p<.01) and traveling (b = 0.92, SE = 0.43, p<.05). Additionally, our results indicated that AD users had higher self-efficacy compared with nonusers (b = 0.40, SE = 0.20, p<.05) after controlling for age, gender, socioeconomic status, as well as physical function level. We suggest that AD provision should be considered in intervention strategies to increase well-being and quality of life in older adults with impaired lower limb function.

